# ChatGPT Knowledge Evaluation in Basic and Clinical Medical Sciences: Multiple Choice Question Examination-Based Performance

**DOI:** 10.3390/healthcare11142046

**Published:** 2023-07-17

**Authors:** Sultan Ayoub Meo, Abeer A. Al-Masri, Metib Alotaibi, Muhammad Zain Sultan Meo, Muhammad Omair Sultan Meo

**Affiliations:** 1 Department of Physiology, College of Medicine, King Saud University, Riyadh 11461, Saudi Arabia; aelmasri@ksu.edu.sa; 2 University Diabetes Unit, Department of Medicine, College of Medicine, King Saud University, Riyadh 11461, Saudi Arabia; ametib@ksu.edu.sa; 3 College of Medicine, Alfaisal University, Riyadh 11533, Saudi Arabia; mzmeo@alfaisal.edu (M.Z.S.M.); momeo@alfaisal.edu (M.O.S.M.)

**Keywords:** ChatGPT, knowledge, intellect level, medical education

## Abstract

The Chatbot Generative Pre-Trained Transformer (ChatGPT) has garnered great attention from the public, academicians and science communities. It responds with appropriate and articulate answers and explanations across various disciplines. For the use of ChatGPT in education, research and healthcare, different perspectives exist with some level of ambiguity around its acceptability and ideal uses. However, the literature is acutely lacking in establishing a link to assess the intellectual levels of ChatGPT in the medical sciences. Therefore, the present study aimed to investigate the knowledge level of ChatGPT in medical education both in basic and clinical medical sciences, multiple-choice question (MCQs) examination-based performance and its impact on the medical examination system. In this study, initially, a subject-wise question bank was established with a pool of multiple-choice questions (MCQs) from various medical textbooks and university examination pools. The research team members carefully reviewed the MCQ contents and ensured that the MCQs were relevant to the subject’s contents. Each question was scenario-based with four sub-stems and had a single correct answer. In this study, 100 MCQs in various disciplines, including basic medical sciences (50 MCQs) and clinical medical sciences (50 MCQs), were randomly selected from the MCQ bank. The MCQs were manually entered one by one, and a fresh ChatGPT session was started for each entry to avoid memory retention bias. The task was given to ChatGPT to assess the response and knowledge level of ChatGPT. The first response obtained was taken as the final response. Based on a pre-determined answer key, scoring was made on a scale of 0 to 1, with zero representing incorrect and one representing the correct answer. The results revealed that out of 100 MCQs in various disciplines of basic and clinical medical sciences, ChatGPT attempted all the MCQs and obtained 37/50 (74%) marks in basic medical sciences and 35/50 (70%) marks in clinical medical sciences, with an overall score of 72/100 (72%) in both basic and clinical medical sciences. It is concluded that ChatGPT obtained a satisfactory score in both basic and clinical medical sciences subjects and demonstrated a degree of understanding and explanation. This study’s findings suggest that ChatGPT may be able to assist medical students and faculty in medical education settings since it has potential as an innovation in the framework of medical sciences and education.

## 1. Introduction

The ‘Chatbot Generative Pre-Trained Transformer (OpenAI’s GPT-3.5)’, developed by OpenAI, is a type of ‘Artificial Intelligence’ (AI) developed to generate dialogues with humans. Algorithms built into the software allow it to recognize natural language inputs and respond with suitable responses. ChatGPT is enriched with advanced technology and techniques to respond to users’ requirements [1]. ChatGPT can swiftly obtain, interpret and provide the required information on a topic. It can compose the information with specified content for a particular person. ChatGPT is a highly capable and effective tool for language translation, article summarization and draft generation, which can be used for various scholarly performances [2].

At the beginning of the year 2023, ChatGPT is gaining popularity among students, faculty, and researchers. However, ChatGPT poses diverse intimidations to the conventional framework of research and education. ChatGPT may develop the option of cheating on online exams and minimize critical thinking skills [3]. ChatGPT can help in drafting various articles and abstracts, summarize the related information and provide suggestions for and generate drafts of any assignments [2]. Text can be written via ChatGPT on an array of subjects in various disciplines [4,5].

### Multiple-Choice Questions (MCQ) and Higher Cognitive Functions

Medical education depends on multiple-choice question (MCQ) pattern examinations while assessing knowledge in various disciplines [6]. MCQs are well accepted and widely used tools in medical education which can promote learning strategies [7]. Worldwide, medical schools and medical licensing examination bodies are conducting MCQ-based examinations. In medical schools, medical students face highly challenging examinations during the various stages of examination in their undergraduate and postgraduate professional careers; hence, medical students study the subject strategically. They look for numerous ways to consolidate their core knowledge and prioritize study resources and approaches that link directly to their examinations [8].

The analytical thinking approach and skills are essential in developing competent physicians. Therefore, worldwide medical educationalists and certifying boards develop MCQs to assess knowledge and skills. Most of the MCQs are written under the higher or lower order levels as per Bloom’s taxonomy. Authors further highlight that better approaches in which students interpret the MCQs would help them better understand and develop clinical reasoning skills [9].

Miller’s pyramid describes the numerous stages of gaining clinical competencies initiated with the learning of the skill. In medical schools, the examination bodies assess the knowledge through various methods, such as essays, short answer questions and MCQs. Higher levels of knowledge acquisition are very important in medical education and can be based on interpretation and application, which can be assessed through medical case presentations, essays and MCQs. The MCQs must be well-designed to assess deep learning. MCQs are an important assessment tool in medical schools [10].

Vegi et al., 2022 [10] conducted a study on MCQs and found that most of students had a positive opinion about MCQ-based examination. Moreover, students preferred that MCQ assess higher-order cognitive skills. Most students expressed their opinion that MCQs should be case scenario-based or conceptual, which tests higher cognitive learning levels. Similarly, Khan and Badr [11] concluded a study and reported that well-constructed MCQs are better than the modified essay in testing higher cognitive skills. It is a fact that medical university assessments enhance higher-order thinking skills and clinical reasoning with substantial knowledge. MCQs are frequently used to evaluate student learning, and well-written MCQs can support the learner in their engagement at higher levels of cognitive reasoning. Bloom’s taxonomy has been used to identify MCQs that can better assess the student’s critical thinking skills and deeper conceptual understanding [12].

MCQs are widely used and reliable assessment tools in various undergraduate and postgraduate medical examinations [13]. MCQs are a valuable way to evaluate the capability of understanding and retention of knowledge, empirical assessment and have distinguished reliability when compared to open-ended question examination. MCQs can assess knowledge, understanding and problem-solving judgmental abilities [14]. Moreover, MCQs are used to assess the various levels of cognitive knowledge, ranging from simple recall of facts at higher-order thinking levels to synthesis, analysis and evaluation [12]. MCQs evaluate the key concepts, facts and principles related to a specific theme and examine the ability to recall and recognize information while selecting the correct answer from multiple options to remember the appropriate knowledge from memory [15,16]. Despite all these advantages, MCQs have some limitations and cannot assess certain skills, such as complex problem-solving skills [17].

Regarding the use of ChatGPT in research, education and healthcare, different perspectives may exist with some level of ambiguity around its acceptability and ideal uses [5]. However, the literature is acutely lacking in establishing a link to assess the intellectual levels of ChatGPT in the medical sciences. Therefore, the present study aimed to investigate the assessment of the knowledge level of ChatGPT in medical education both in basic and medical science examination-based performance in order to investigate its impact on the medical examination system. This study’s findings may provide insight and benefit medical students and faculty in medical education settings since it has potential as an innovation in the medical sciences and the education framework.

## 2. Research Methodology

### 2.1. Study Design and Settings

The present cross-sectional study was conducted in the Department of Physiology, College of Medicine, King Saud University, Riyadh, Saudi Arabia in May 2023.

### 2.2. Establishment of Multiple-Choice Question Bank

The research team members prepared the MCQ bank based on the information and questions from various textbooks, including Guyton and Hall’s *Text of Medical Physiology*; Snell’s *Clinical Anatomy by Regions*; *First Aid USMLE Step1*; *First Aid USMLE 2*; *AMBOSS Step 1*, Kumar and Clark’s *Clinical Medicine*; and the university examination pools. Initially, a subject-wise question bank was established with a pool of MCQs. The research team members carefully reviewed the MCQs’ contents and assured that the MCQs were relevant to the subject contents and appropriately challenging. Each question was scenario-based with four sub-stems and had a single correct answer. The MCQ scenario was modified where necessary without disturbing the exact meaning of the question and key answer.

MCQs were evaluated for quality, and it was ensured that the MCQs were unambiguous, with only one correct answer. The language was simple and easy to understand. The investigator team members also proofread the MCQs for any errors, typos, confusing or misleading statements or inconsistencies. It was also checked that the options were well constructed and that there were no clear hints or clues within the questions. Moreover, a pilot test was performed on ChatGPT with five MCQs to check the technicality of the selected MCQs; these five MCQs were excluded from the final testing exam pool. Once the investigators were satisfied with the preparation of the MCQ pool and its quality, all questions were compiled into the final exam format.

### 2.3. Selection of Multiple-Choice Questions

After the preparation of the MCQ bank, 100 Multiple Choice Questions (MCQs) in various disciplines of basic medical sciences (50 MCQs) and clinical medical sciences (50 MCQs) were randomly selected from the MCQ bank from the pool of questions in each subject category. Out of 100 MCQs in various disciplines of basic and clinical medical sciences, 50 MCQs were selected from basic medical sciences, anatomy (10), biochemistry (10), physiology (10), pathology (10) and pharmacology (10). And 50 MCQS were selected from clinical medical sciences, including medicine (10), surgery (10), gynecology and obstetrics (10), ophthalmology (10) and ear, nose and throat (10). The research team members from basic and clinical medical sciences carefully checked all the MCQs, MCQ scenarios and their answer keys.

### 2.4. Examples of Multiple-Choice Questions

The MCQ pattern was to ask questions as per the required standards in medical education. “What would be the most appropriate patient’s diagnosis based on the information provided?” or “Which one of the following is the most appropriate treatment option?” “Which one of the following best represents the most appropriate next step in management?” or “The patient’s condition is most probably caused by which of the following pathogens?”. The textual questions were scored according to the number of correct answers. Chart question scores were determined by multiplying the number and the correct rate of text questions.

### 2.5. Exclusion of MCQS

**Figure 1:**
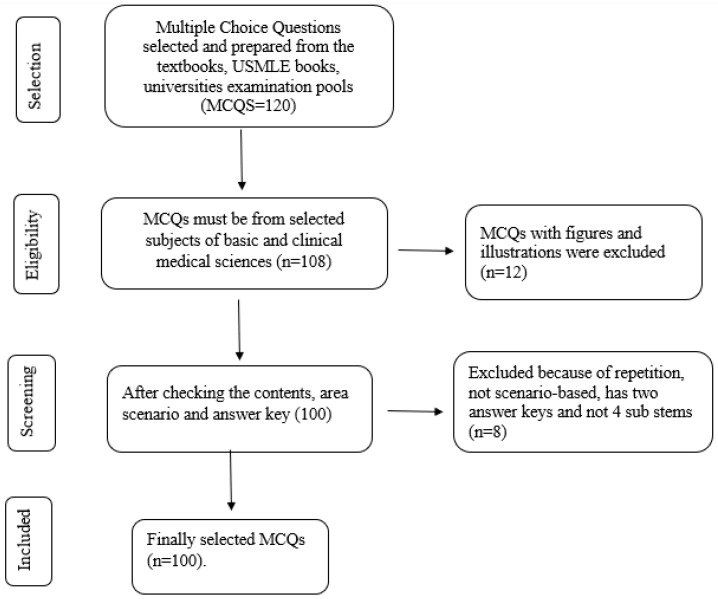
Flow diagram of the selection of MCQs.

The MCQs which were not related to the specific subject area of basic and clinical medical sciences, with misleading stems, repetition, without appropriate scenarios and with biased answer keys were excluded from the study. We also removed the test MCQs that had visual components like clinical images, graphs, and illustrations from our study, as it is not possible to insert the figure or illustration in the given area in ChatGPT. Moreover, ChatGPT is unable to scan or appropriately read the clinical images, graphs, and illustrations; hence, we excluded these MCQs from the study (Figure 1). Five MCQs which were used for the technical checking in the ChatGPT were also excluded from the examination pool.

### 2.6. Entry of Multiple-Choice Questions into the ChatGPT

**Figure 2:**
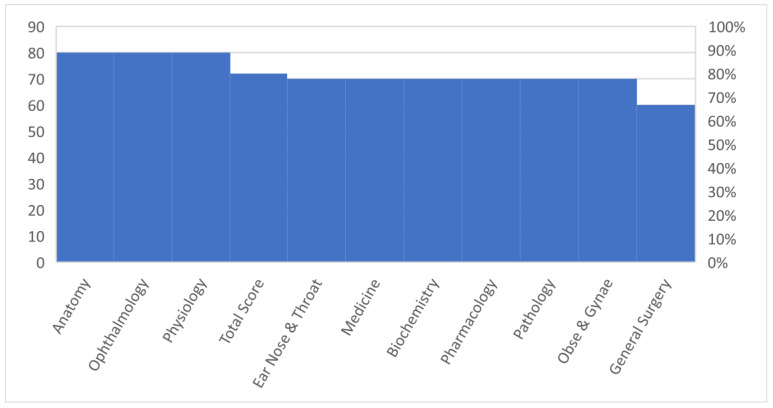
Marks obtained by ChatGPT in various basic and clinical medical science subjects.

**Figure 3:**
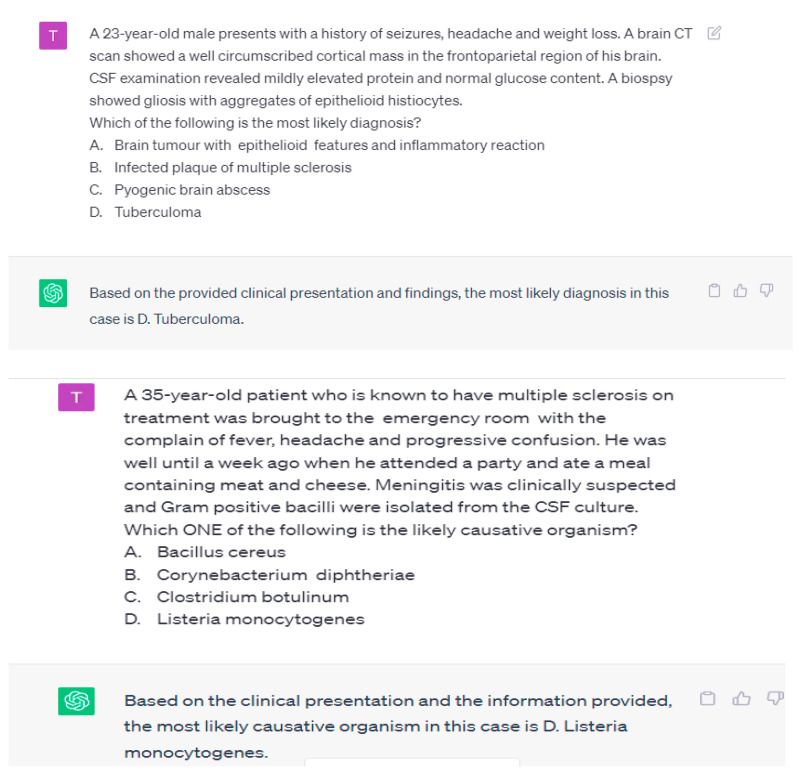
The pattern of MCQs used to assess the knowledge of ChatGPT 3.5 in basic and clinical medical science subjects.

The task was given to ChatGPT to assess the response and knowledge level of ChatGPT (Table 1 and Table 2, Figure 2). The MCQs were entered manually one by one, and a fresh ChatGPT session was started for each entry to avoid memory retention bias (Figure 3).

### 2.7. Data Collection and Score System

The questions were used as input in ChatGPT, and the responses that the tool gave were transcribed and stored in a separate file. The MCQs asked the reasoning behind choosing whichever answer ChatGPT thought was right. The first response that was obtained was taken as the final response, and we did not use the choice of “regenerate response”. Based on a pre-determined answer key, scoring was executed on a scale of 0 to 1, with zero representing incorrect and one representing correct.

### 2.8. Ethical Approval and Statistical Analysis

ChatGPT is currently a free, open-source online tool that is accessible to users with a registration on the website, and all information that was collected was executed from its most recent version (version 3.5 as of May 2023). The MCQ bank was established from the textbook sources and university examination pools, and, since the study did not directly involve any animal or human participants, ethical approval was not required. The data were carefully reviewed and findings were recorded and analyzed. The analysis was based on each question and its response. The data were expressed as numbers and percentages and analyzed using descriptive statistical tests.

## 3. Results

The knowledge of ChatGPT was evaluated for this study, and its ability to provide the response on individual MCQs in basic and clinical medical sciences was investigated. The questions were based on a high-stakes, comprehensive standardized testing question which covered diverse topics in clinical and basic medical sciences. The levels of standardization and regulation in question difficulty and complexity made it an excellent input substrate to test the ChatGPT. The MCQ-based examination was well organized and well arranged.

The MCQs were based on the subject discipline in basic medical sciences and clinical medical science. A total of 50 MCQs were selected from basic medical sciences, anatomy (10), biochemistry (10), physiology (10), pathology (10) and pharmacology (10), and 50 MCQs were selected from clinical medical sciences, medicine (10), surgery (10), gynecology and obstetrics (10), ophthalmology (10) and ear, nose and throat (10) (Table 1).

Table 2 shows ChatGPT’s knowledge of the set of MCQs assessed. The scores for MCQs are shown in Table 2. Out of 100 MCQs in various disciplines of basic and clinical medical sciences, ChatGPT attempted all the MCQs and obtained 37/50 (74%) marks in basic medical sciences and 35/50 (70%) marks in clinical medical sciences, with an overall score of 72/100 (72%) in both basic and clinical medical sciences. ChatGPT performed more accurately on basic medical science questions with 74% (37/50) as compared to clinical science questions with 70% (35/50) (Table 2, Figure 2).

## 4. Discussion

ChatGPT has garnered great attention from the public, students, academicians, researchers and science communities. ChatGPT swiftly responds with appropriate and articulate answers across the various disciplines of subjects. ChatGPT is a useful tool to enhance scientific knowledge, generate an essay and provide explanations. There is great debate among the public and academicians about the knowledge and intelligence level of ChatGPT. The present study aimed to investigate the assessment of the knowledge level of ChatGPT in basic and clinical medical sciences and medical education. The present study results reveal that ChatGPT answered and obtained good grades in both basic and clinical science examinations. ChatGPT is designed to generate human-like responses and engage the users in conversational interactions and rapid responses within seconds. ChatGPT is guided by a wide range of internet text data, which allows it to understand and produce text in a variety of contexts. It can answer questions, provide explanations, offer suggestions, create conversational dialogues and assist with multiple tasks [18]. Limited studies are available to investigate the medical education examination-based performance of ChatGPT.

Passby et al., 2023 [19] conducted a study on a ChatGPT and recorded the score of answering multiple-choice questions. ChatGPT-3.5 achieved an overall score of 63.1%, and ChatGPT-4 scored 90.5%, significantly higher. Hence, ChatGPT-4 can answer clinical questions and achieve a passing grade.

Duong et al., 2023 [20] assessed the performance of ChatGPT versus human respondents in answering MCQs about human genetics and its aspects. The performance of ChatGPT did not vary significantly from human respondents. In comparison to the 66.6% accuracy of human respondents, ChatGPT achieved a score of 68.2%. As for memorization-type questions, humans and ChatGPT both got reasonable results compared to critical thinking questions. When given the same question repeatedly, ChatGPT would frequently give different answers (16% of the initial responses), which included both initially correct and incorrect answers and gave explanations for both incorrect and correct answers. Despite its impressive performance, ChatGPT currently displays significant shortcomings for either clinical or other high-stakes applications.

Wang et al., 2023 [21] examined the accuracy of ChatGPT on the Taiwanese Pharmacist Licensing Examination and investigated its potential role in pharmacy education. The authors reported that the correct rate of ChatGPT in Chinese and English questions was 54.4% and 56.9% in the first stage and 53.8% and 67.6% in the second stage. On the Chinese test, only the pharmacology and pharmaco-chemistry sections received passing scores. The English test scores were higher than the Chinese test scores across all subjects and were significantly higher in dispensing pharmacy and clinical pharmacy as well as therapeutics. The authors conclude that ChatGPT 3.5 failed the Taiwanese Pharmacist Licensing Examination. It can be improved quickly through deep learning. Similarly, Suchman et al., 2023 [22] assessed the performance of ChatGPT-3 and ChatGPT-4 based on the questions and answers of ‘The American College of Gastroenterology Self-Assessment Tests’. In both versions of ChatGPT, exact questions were entered. The Overall ChatGPT-3 scored 65.1% and GPT-4 scored 62.4%. It was concluded that ChatGPT did not pass the ACG self-assessment test, and the authors did not recommend its use for medical education in gastroenterology in its current form.

Humar et al., 2023 [23] evaluated ChatGPT’s performance on the Plastic Surgery In-Service examination and compared it to the residents’ performance. The authors selected a pool of questions out of which ChatGPT answered 55.8% correctly. Additionally, ChatGPT scored the highest on the 2021 exam (a score of 60.1%) with 58.7% on its comprehensive part. On the 2022 In-Service examination, 57% of the questions were answered correctly. As a conclusion, ChatGPT’s performance was equivalent to that of a first-year resident. However, in comparison to residents with superior levels of training, ChatGPT’s performance was poor. While ChatGPT has numerous evident benefits and prospective applications in healthcare and medical education, further studies are needed to determine its efficacy.

Gupta et al., 2023 [24] tested ChatGPT’s accuracy on the examination ‘Plastic Surgery Inservice Training Examination (PSITE)’ to check if it can be used as a tool in resident education. ChatGPT answered 54.96% of correct questions and incorporated logical reasoning in 88.8% of questions. ChatGPT is a versatile tool that has the potential to impact resident education by providing general knowledge, providing case-based learning, clarifying information and promoting evidence-based medicine.

Gilson et al., 2023 [25] evaluated ChatGPT’s performance on questions which were within the bounds of the United States Medical Licensing Examination Step 1 and 2 examinations. Two sets of multiple-choice questions were used for evaluating ChatGPT’s performance, having questions for both Steps 1 and 2. AMBOSS, a renowned question bank used by medical students, was used for deriving questions for the first set, with the second set from the National Board of Medical Examiners (NBME). ChatGPT achieved accuracies of 44% (44/100), 42% (42/100), 64.4% (56/87) and 57.8% (59/102), respectively. The performance exhibited a significant drop as questions became more difficult within the AMBOSS-Step1 dataset. The model achieves a passing score for a third-year medical student by surpassing the 60% threshold on the NBME-Free-Step-1 dataset. The evidence considered suggests that ChatGPT offers great potential as an interactive medical education tool to help people learn.

Previous work in medical question-answering tends to focus on specific tasks with the model’s performance being prioritized over generalizability on a dataset of questions collected from Chinese medical licensing exams; Jin et al. [26] attained an accuracy of 36.7%. Likewise, Ha et al. [27] reported an accuracy of 29% on USMLE Step 1 and 2 questions. Therefore, ChatGPT represents an important step forward in distinct areas by expanding beyond a simple question-and-answer function. The first is generalizability, the second front is accuracy and the third is ChatGPT, which marks the greatest jump forward in user interpretability due to its conversational interface. As we have seen, each response includes some reasoning, and the option to ask follow-up questions enables the user to have a more comprehensive understanding of the concept rather than simply one answer output.

Das et al., 2023 [28] reported that ChatGPT scored an accuracy of about 80% in microbiology, thus answering questions on both first- and second-order knowledge. The findings of the study suggest that ChatGPT can be a powerful tool for automated question-answering within the area of microbiology. Nevertheless, to increase their performance and make them appropriate for academic use, language models must continue to undergo training and development improvements.

Huh et al., 2023 [29] compared ChatGPT’s interpretation skills and its knowledge with medical students in Korea through a parasitology examination containing 79 items. The performance of ChatGPT was poorer than that of medical students, and its correct answer rate was unrelated to the items’ knowledge level. The knowledge and interpretation skills of ChatGPT for this exam were not comparable to medical students in Korea.

Ghosh et al., 2023 [30] conducted a study which highlighted the potential of ChatGPT to evolve into a successful tool for answering and understanding medical biochemistry questions, including those that require higher-order thinking, with an obtained median score of 4/5. Regardless, the study highlights the recent advances in the ever-growing field of medical sciences and academics; ChatGPT will still need continuous training to further improve its performance and make it unlock its full potential. Sinha et al., 2023 [31] reported an accuracy score of about 80% for ChatGPT solving higher-order reasoning questions related to pathology. Therefore, students and academicians can obtain help from it for solving such questions. However, again, further studies are needed to reach better and more reliable accuracy scores. The literature raises some concerns about its role in diagnostic purposes without human expert oversight [32].

ChatGPT attempted the MCQs and attained satisfactory grades. This groundbreaking AI tool opens multiple doors for questioning that develops the foundation for the knowledge which is needed to justify underlying medical reasonings. More studies in the literature are required to further analyze and evaluate the capability levels of ChatGPT in attempting medical-reasoning questions. Novel medical educational methods could also be developed that make full use of the potential of ChatGPT as technology further advances.

In this study, the MCQ examination covered a broad range of topics within the subject area of basic and clinical medical sciences and performed a comprehensive assessment. The grades obtained by ChatGPT demonstrate that it has critical thinking and problem-solving abilities. MCQs can evaluate the higher-order thinking skills in the complex situations required while analyzing information and apply logical reasoning and decisions when selecting the most appropriate answer choice. It’s important to note that while MCQs are valuable in assessing knowledge, they may not capture all aspects of understanding the practical application of assessment of practical exams or performance-based evaluations, which is necessary for a comprehensive evaluation of skills. As we did not assess the skill abilities, future studies must be conducted to assess the role of ChatGPT in the assessment of skills in addition to knowledge.

In this study, ChatGPT correctly selected the option from multiple choice options; it shows that it can exclude the distractors, which are incorrect answer choices that resemble the correct answer. This feature assesses the depth of knowledge and the ability to differentiate between similar concepts and identify misconceptions or gaps in understanding. It shows that ChatGPT knows the different sub-topics with a comprehensive understanding and higher-order thinking and problem-solving abilities of the subject matter in basic and clinical medical sciences. The implications of the findings suggest that ChatGPT can be a good tool and source of knowledge for medical staff and student; however, practical application of this software must be carefully regulated since the scores of AI software on examination questions designed for humans may give rise to the need of a change in the future medical examination system.

### Study Strengths and Limitations

The strengths of this study are the following: This is the first novel study that assessed the knowledge level of ChatGPT in various subjects of basic and clinical medical sciences. MCQs were selected from the various textbooks and a university pool of questions and were checked by two faculty members to maintain the quality of the question. Similar to any study, this study has some limitations. The analysis was limited by the 100 MCQ size; for instance, it categorized ChatGPT’s output according to subject taxonomy and 10 MCQs from each discipline in various basic and clinical medical science subjects. Future large sample-sized studies should be conducted. Moreover, with the passage of time and the advancement of technology, these AI tools could further develop and therefore results may be further improved. Finally, the tool must be assessed in both real-world and controlled situations with students throughout the engagement and knowledge range to truly evaluate its utility.

## 5. Conclusions

The ChatGPT obtained a satisfactory score in basic and clinical medical science examinations. It has the potential for answering MCQs and demonstrated a degree of understanding and reasoning explanations for answers. ChatGPT might be able to assist in medical education, in both basic and clinical medical sciences. Therefore, this marks a major achievement in the field of artificial intelligence and its development. Overall, this study’s findings suggest that ChatGPT may be able to assist medical students and faculty in medical education settings since it has the potential as an innovation in the framework of medical sciences and education. Similar studies within various domains in medical sciences should be conducted to reach better conclusions.

## Figures and Tables

**Table 1:** Distribution of MCQs in basic and clinical medical sciences.

Basic Science MCQs	Number of MCQs
Anatomy	10
Physiology	10
Biochemistry	10
Pharmacology	10
Pathology	10
**Clinical Science MCQs**	
Medicine	10
General surgery	10
Gynecology and obstetrics	10
Ophthalmology	10
Ear, nose and throat	10
Total MCQs	100

**Table 2:** Marks obtained by ChatGPT.

Basic Science MCQs	Marks Obtained
Anatomy	8/10 (80%)
Physiology	8/10 (70%)
Biochemistry	7/10 (60%)
Pharmacology	7/10 (60%)
Pathology	7/10 (60%)
Subtotal score	37/50 (74%)
**Clinical Science MCQs**	
Medicine	7/10 (70%)
General surgery	6/10 (60%)
Gynecology and obstetrics	7/10 (60%)
Ophthalmology	8 (80%)
Ear, nose and throat	7 (70%)
Subtotal score	35/50 (70%)
Total score	72/100 (72%)

## Data Availability

Not applicable.
